# Electronic and Conventional Cigarette Use and Academic Achievement Among Predominantly Low-Income Black High School Students

**DOI:** 10.3390/children12091237

**Published:** 2025-09-16

**Authors:** Payam Sheikhattari, Rifath Ara Alam Barsha, Chidubem Egboluche, Shervin Assari

**Affiliations:** 1 School of Community Health and Policy, Morgan State University, Baltimore, MD 21251, USAchegb4@morgan.edu (C.E.); 2 John D. Bower School of Population Health, University of Mississippi Medical Center, Jackson, MS 39216, USA; rbarsha@umc.edu; 3 Department of Internal Medicine, Charles R Drew University of Medicine and Science, Los Angeles, CA 90059, USA

**Keywords:** adolescents, tobacco use, e-cigarettes, academic performance, school performance, urban youth, health disparities, Baltimore City, cross-sectional study

## Abstract

Background: Tobacco use remains a major public health concern among adolescents, particularly as electronic cigarette (e-cigarette) use has increased in recent years. While academic performance has been linked to health-risk behaviors, less is known about its specific relationship to tobacco use among predominantly Black youth in urban settings. Understanding this association is essential for informing targeted prevention strategies. Objective: To examine the association between academic performance (self-reported grades from A to F) and use of tobacco products—including conventional cigarettes, e-cigarettes, and other forms—among predominantly Black high school students in Baltimore, a city marked by socioeconomic and health disparities. Methods: A cross-sectional analysis was conducted using survey data from 604 public high school students in Baltimore. The main predictor was self-reported average school grades. Outcomes included ever use of (1) combustible tobacco products, (2) e-cigarettes, and (3) any tobacco product. Logistic regression models estimated associations between academic performance and each tobacco outcome, adjusting for age, sex, race, parental education and employment, and household structure. Results: Among participants (mean age = 16.1 years), 20.2% reported using e-cigarettes, 7.1% used cigarettes, and 25.2% had used any tobacco product. Academic performance was inversely associated with all forms of tobacco use. Students with A, B, or C grades had significantly lower odds of e-cigarette use compared to those with D or F grades. Students with A grades had lower odds of cigarette use and any tobacco use. Conclusions: Lower academic achievement was consistently associated with higher odds of tobacco use among predominantly Black adolescents. Academic performance may help identify youth at higher risk of tobacco use and guide school-based prevention and intervention strategies in similar urban settings.

## 1. Introduction

Tobacco use is a leading preventable cause of death in the United States [1]. Most individuals who use tobacco start during adolescence [2]. Although youth cigarette smoking has declined over the past two decades [3], the rapid rise in electronic cigarette (e-cigarette) use presents new public health challenges [4,5]. Adolescents who use tobacco—via combustible cigarettes, e-cigarettes, or other products—face increased risk of nicotine dependence, respiratory problems, academic difficulties, and continued substance use into adulthood [6].

Understanding the social and behavioral determinants of tobacco use during adolescence is particularly important in communities affected by structural disadvantage [7,8]. Baltimore, Maryland, exemplifies how intersecting health and socioeconomic inequities shape adolescent life trajectories. Urban centers such as Baltimore have long struggled with overlapping health and socioeconomic inequities [9,10]. Racial and economic segregation, under-resourced schools, and chronic community stress disproportionately affect Black youth in these settings [11]. While not all students in these communities live in poverty, many attend schools serving predominantly low-income populations where the burden of targeted tobacco marketing, stress exposure, and limited access to cessation resources is elevated [12,13].

Academic performance is a key social determinant of adolescent health [14]. Lower school grades have been linked to a range of negative outcomes, including higher rates of behavioral health concerns, lower educational attainment, and greater engagement in health-compromising behaviors such as tobacco and substance use [15]. Although prior studies show an inverse relationship between academic performance and cigarette use [16], fewer have examined newer tobacco products like e-cigarettes or examined these associations among predominantly Black youth in urban environments.

Importantly, the relationship between academic performance and tobacco use may not be uniform across all product types [17,18]. E-cigarettes are often viewed as more socially acceptable and less harmful than cigarettes and may appeal to adolescents across a broader academic spectrum [19]. Conversely, cigarette smoking has historically been more stigmatized and more strongly associated with school disengagement and poor academic outcomes. Thus, academic achievement may differentially relate to use of e-cigarettes versus combustible tobacco, with the strength or direction of associations varying by product. Analyzing tobacco types separately may offer a more nuanced understanding of how academic performance functions as a protective—or, in some cases, less protective—factor depending on the context and substance [17].

In addition to academic stress, many Black adolescents in urban public schools confront layered risk factors that shape both educational experiences and health behaviors [20,21]. These include exposure to racial discrimination, community violence, economic insecurity, and limited access to culturally affirming supportive resources [22,23]. Such conditions may not only elevate the likelihood of using tobacco as a coping mechanism but may also reduce the extent to which academic success can buffer against risk behaviors [24]. Understanding how these contextual factors intersect with school performance and tobacco use is critical to advancing equity in prevention science [8,23].

In recent years, e-cigarette use has emerged as a growing public health concern [25], particularly among underserved youth in urban settings [26,27]. Low-income urban populations face multiple structural disadvantages, such as targeted marketing, limited cessation resources, and normalized vaping environments, that increase vulnerability to early and sustained use [28]. Despite declining rates of combustible cigarette use among adolescents, the rising prevalence of e-cigarettes, especially in low-income, racially segregated urban neighborhoods, signals a shifting landscape of nicotine exposure and addiction risk [29,30]. Addressing this trend is especially urgent given the potential long-term health consequences and the lack of tailored prevention strategies for marginalized low-income urban communities. Highlighting e-cigarette use in this context not only reinforces the timeliness of this study but also positions it to contribute meaningfully to health equity research and intervention efforts.

Recent findings continue to expand our understanding of adolescent tobacco use. PATH Wave 5 analyses [31,32,33,34,35,36,37] show how race/ethnicity, sex, sexual orientation, SES, and policy shape electronic and conventional tobacco use in general populations and marginalized populations [31,32]. In the PATH study, a recent study showed three latent classes were identified. The “normative” class reported low prevalence of all symptoms, the “severe internalizing and non-violent externalizing” class reported severe internalizing problems and non-violent externalizing problems, and the “severe” class reported high prevalence of all symptoms. Tobacco use was highest for the “severe” class and lowest for the “normative” class across products [38]. Randomized controlled trials (RCTs) of school-based interventions—particularly those incorporating social–emotional learning (SEL)—have demonstrated promising reductions in vaping and tobacco use susceptibility and initiation [39]. These findings underscore the importance of situating this study within a broader evidence base and demonstrate the potential for school-centered efforts—like the CEASE program—to serve as effective platforms for both education and prevention.

These findings are consistent with longitudinal work by Dearfield et al. [40]. They conducted a study to explore whether starting to use e-cigarettes is linked to later academic performance among adolescents in the United States. Using data from Waves 2 through 4 of the youth and parent surveys from the Population Assessment of Tobacco and Health (PATH) study, their analysis focused on youth aged 12 to 15 who had never used tobacco at Wave 2 (*n* = 4960). E-cigarette and cigarette initiation were identified at Wave 3, and academic performance outcomes were assessed at Wave 4. Weighted multivariable linear regression models were used to examine the associations, adjusting for baseline characteristics measured at Wave 2. By Wave 3, 4.3% of participants had initiated e-cigarette use and 1.9% had started using cigarettes. Those who began using e-cigarettes at Wave 3 showed lower academic performance one wave later compared to their peers who had not initiated use (adjusted regression coefficient [ARC] = −0.22; 95% CI: −0.43, −0.02). A similar, and even stronger, negative association was found for youth who initiated cigarette smoking (ARC = −0.51; 95% CI: −0.84, −0.18). Their findings suggested that initiating e-cigarette or cigarette use during early adolescence may be linked to poorer academic outcomes, reinforcing the need for prevention strategies that address both academic and health risks [40].

A major strength of this study is its integration within the CEASE (Communities Engaged and Advocating for Smoke-Free Environments) initiative, which enhances its translational potential. CEASE is a long-standing, community-engaged model for tobacco prevention that incorporates school-based, family-centered, and policy-level strategies. Interpreting the current findings within this established public health framework increases their relevance for intervention design—especially in urban school settings that serve racially and economically marginalized populations.

## 2. Objective

This study aims to examine the association between academic performance and the use of conventional cigarettes and electronic cigarettes (e-cigarettes) among high school students in Baltimore. The sample includes students from public schools in predominantly Black, urban neighborhoods with varying levels of socioeconomic disadvantage. By assessing how school grades relate to specific forms of tobacco use, this study seeks to inform more targeted, equitable, and developmentally appropriate interventions that address the intertwined educational and behavioral health needs of marginalized urban youth.

## 3. Methods

### 3.1. Design and Setting

This study employed a cross-sectional design, drawing on data from the CEASE Youth: School Survey—an online instrument distributed among high school students in Baltimore City. The survey was developed through a partnership between the CEASE program at Morgan State University and the American Lung Association’s Not On Tobacco (N-O-T) initiative. It was implemented in selected public high schools throughout the city to gather information on tobacco use, mental health, and associated behavioral and demographic variables. Students completed the survey privately and independently, which supported wide participation and helped reduce the risk of biased reporting.

### 3.2. CEASE Youth Survey

A brief overview of the CEASE Youth Survey’s structure and administration is provided here. The survey was completed by students electronically in a self-guided format. It began with an informed consent process, followed by the entry of general, non-identifiable details such as the survey date, school name, a four-character code derived from the respondent’s initials and phone number, and their birth month and year. To maintain confidentiality, no personal identifiers or names were recorded. As a gesture of appreciation, participants received a USD 10 electronic gift card after completing the survey.

### 3.3. CEASE Initiative

This study was conducted as part of the *Communities Engaged and Advocating for a Smoke-free Environment* (CEASE) research initiative. CEASE is a community-based participatory research (CBPR) program rooted in Baltimore, developed through a partnership between Morgan State University and local residents. It was created in response to persistently high smoking rates in underserved, predominantly Black urban neighborhoods, particularly those marked by socioeconomic disadvantages. The program is grounded in principles of community empowerment, peer leadership, and culturally responsive intervention strategies aimed at supporting individuals in adopting and sustaining smoke-free lifestyles. CEASE encompasses both research and intervention components. While the initiative conducts community-based surveys across age groups, it also delivers evidence-informed tobacco cessation interventions. The CEASE survey component includes collaborations with organizations such as the American Lung Association and the Baltimore City Public School System to conduct epidemiological assessments of tobacco use, particularly among students. On the intervention side, CEASE offers free group-based cessation services delivered in person, online, or through hybrid formats. These services combine behavioral counseling, nicotine replacement therapy (NRT), and health education. A defining element of CEASE is its peer-led model, in which trained peer motivators—often former smokers from the same communities—facilitate sessions. This approach enhances cultural relevance and fosters trust, making participants more likely to engage and persist in their quit attempts. Over the years, CEASE has evolved through multiple phases, expanding its reach and refining its methods in response to community feedback and evaluation data. For example, the program has implemented 12-week, 4-week, and 2-week cessation curricula, which typically involve weekly group meetings and provide both social and material support. Quit rates have been promising, with some cohorts reporting abstinence rates as high as 25% in 12 weeks. These outcomes are particularly meaningful given the program’s focus on marginalized populations who often encounter barriers to accessing traditional healthcare services. To increase accessibility and scalability, CEASE has developed shorter, more flexible program formats, including one- and four-session models. The initiative also introduced the innovative “Monday-Enhanced” model, which incorporates behavioral science by scheduling quit-related activities and messaging on Mondays—capitalizing on the psychological tendency for individuals to initiate behavior change at the beginning of the week. In addition to its intervention work, CEASE continues to monitor patterns of tobacco use, including emerging products such as e-cigarettes, among both youth and adults. Collectively, CEASE exemplifies the value of locally tailored, theory-driven, and community-led approaches to reducing tobacco-related disparities in historically underserved urban populations.

### 3.4. Survey Instrument

The survey instrument covered several core domains, including demographic characteristics, socioeconomic indicators, academic performance, tobacco use behaviors, attitudes and beliefs about tobacco, and related health and behavioral factors. Participants were asked to report their age, gender identity, and racial/ethnic background. Socioeconomic status was assessed through items on parental education, parental employment, household income (measured in categorical ranges), and household composition, including whether the student lived in a single- or two-parent home.

Academic performance was evaluated by asking students to report their current grade level and their perceived average grades over the past academic year. Grades were reported using a traditional scale ranging from A to F. We combined D and F grades due to the relatively small number of students reporting F grades, which limited the statistical power to model them separately.

Tobacco and nicotine product familiarity and use were also assessed. Participants indicated whether they had heard of various tobacco products, including conventional cigarettes, cigars, smokeless tobacco, and electronic cigarettes. They were then asked a series of belief and attitude questions related to the perceived health risks, addictiveness, and chemical contents of these products. These items used a four-point Likert scale ranging from “strongly agree” to “strongly disagree.”

Tobacco use was measured by asking whether participants had ever used conventional cigarettes, e-cigarettes, or other products, even once or twice. Lifetime and recent use were assessed for each product. Participants also reported whether a healthcare provider had advised them to quit using tobacco or e-cigarettes in the past year.

To capture co-occurring risk behaviors, participants were asked whether they had ever consumed alcohol—even just a few sips—or used marijuana. Awareness of local tobacco cessation resources was also assessed, with students indicating whether they knew of any school- or community-based cessation programs (response options: yes, no, not sure).

The survey included items on media exposure, asking participants how frequently they encountered tobacco advertisements or promotions across various platforms, including social media, streaming services, retail stores, and printed materials. Students also reported whether they had received coupons or discount offers for tobacco products in the past year.

Technology use was captured by asking students which digital platforms they regularly used for communication, including social media, messaging apps, and email. Lastly, the survey assessed general health status using a four-point scale (excellent, good, fair, poor) and included items on physical and mental health. Students were asked to report any cognitive difficulties and to indicate how frequently they experienced stress, anxiety, or depressive symptoms during the past 30 days.

### 3.5. Variables

Academic Performance (Grades): Academic performance was assessed using a self-reported survey question, “During the past 12 months, how would you describe your grades in school?”, with response options: *Mostly A’s*, *Mostly B’s*, *Mostly C’s*, *Mostly D’s*, and *Mostly F’s*. For analysis, responses were categorized into four groups: A, B, C, and D & F.

Ever Conventional Cigarette Use: This variable was based on a single self-report item asking whether the respondent had ever tried a conventional cigarette, even once or twice. Responses were coded dichotomously (yes/no).

Ever E-Cigarette Use: E-cigarette use was defined by a parallel item asking whether the respondent had ever tried an electronic cigarette, even once or twice. Responses were coded as yes or no.

Ever Use of Any Tobacco Products: This variable was defined as whether they had ever used electronic cigarettes, conventional cigarettes, or any other tobacco products. This variable was also treated as binary.

Covariates: Demographic and socioeconomic covariates included age, gender, race, parental employment status, household composition (one-parent vs. two-parent household), and parent or guardian educational attainment.

This study received Institutional Review Board (IRB) approval from Morgan State University (IRB00008794). Active parental consent and minor assent were obtained for all participants prior to survey administration. In alignment with ethical standards for research with minors, a waiver of parental consent was not requested nor granted. The survey was administered anonymously to reduce social desirability bias, although this bias remains a consideration given the sensitivity of questions regarding substance use.

### 3.6. Statistical Analysis

Descriptive statistics were first calculated to summarize sample characteristics and the distribution of all key variables overall and by academic performance (grade). A series of unadjusted and adjusted logistic regression models were then used to examine the association between academic performance and each form of tobacco use, conventional cigarettes, e-cigarettes, and any tobacco use. All models controlled for the demographic and socioeconomic variables described above. Results are presented as Odds Ratios (ORs) with corresponding 95% Confidence Intervals (CIs) and *p*-values.

### 3.7. Data Use and Confidentiality

All survey data were collected and analyzed in a de-identified format to protect participant confidentiality. Results are reported in aggregate and are being used to evaluate the CEASE-NOT program. The findings will help guide the development of targeted, evidence-based interventions aimed at reducing tobacco use and associated health risks among youth in Baltimore City.

### 3.8. Institutional Review Board (IRB)

This study received ethical approval from the appropriate Institutional Review Board. All participants provided informed assent before beginning the survey.

## 4. Results

### 4.1. Descriptive Data

Table 1 presents the descriptive statistics of the study sample. The analytic sample consisted of 604 adolescents with a mean age of 16.1 years (Standard Deviation [SD] = 1.5). Among the participants, 20.2% reported ever using e-cigarettes, 7.1% reported ever using conventional cigarettes, and 25.2% reported ever using any tobacco product. The sample was predominantly female (54.0%), and the majority of participants identified as Black (88.3%). In terms of academic performance, 45.0% of adolescents reported earning mostly B grades, followed by 26.7% with A grades, 24.7% with C grades, and 3.6% with D or F grades. Regarding parental educational attainment, 35.6% reported that their parent or guardian had completed high school, 21.4% had some college education, 17.2% had a graduate degree or higher, 12.9% had completed college, and 12.4% had less than a high school diploma. Most participants (85.3%) reported having at least one employed parent or guardian. Additionally, 37.2% of adolescents lived in a two-parent household, while 62.6% lived in a single-parent household.

### 4.2. Bivariate Associations

Table 2 presents the distribution of tobacco use and sociodemographic characteristics by academic performance. The prevalence of ever using electronic cigarettes differed significantly across grade groups (*p* < 0.001). Among students who reported ever using electronic cigarettes, the largest proportion were those earning B grades (42.6%), followed by those with C grades (30.3%), A grades (18.0%), and D or F grades (9.0%). A similar pattern was observed for ever use of any tobacco product, with the highest proportion of users among students with B grades (42.1%), followed by those with C grades (34.2%), A grades (16.5%), and D or F grades (7.2%) (*p* < 0.001). For conventional cigarette use, the majority of users were also among students with B grades (51.2%), followed by those with C grades (30.2%), D or F grades (9.3%), and A grades (9.3%) (*p* < 0.05).

Gender distribution was relatively balanced across academic performance groups. White students were more likely to be represented in the A-grade group (50.0%), while students identifying with other racial backgrounds were more concentrated in the B-grade group (54.9%). Parental education level was significantly associated with academic performance (*p* < 0.05). Students whose parents had a graduate degree or higher were most frequently in the B-grade group (53.8%), whereas those whose parents had less than a high school diploma were primarily concentrated in the B- (53.3%) and C- (25.4%) grade categories. Average age also differed by academic performance (*p* < 0.05), with students earning D or F grades being the oldest on average (mean age = 16.8 years).

### 4.3. Multivariable Analysis

Table 3 shows logistic regression results for ever use of electronic cigarettes. In both unadjusted and adjusted logistic regression models, compared to students with D and F grades, those with higher academic performance had significantly lower odds of ever using electronic cigarettes. Students with C grades had lower odds (Adjusted Odds Ratio [AOR] = 0.38; *p* < 0.05), followed by those with B grades (AOR = 0.29; *p* < 0.05) and A grades (AOR = 0.19; *p* < 0.05). Regarding parent or guardian educational attainment, adolescents whose parents held a graduate degree or higher had significantly lower odds of electronic cigarette use in the unadjusted model (Odds Ratio [OR] = 0.47; *p* < 0.05), but this association did not remain significant after adjustment. Living in a two-parent household was also associated with significantly lower odds of electronic cigarette use in the unadjusted model (OR = 0.65; *p* < 0.05), although this association was no longer statistically significant in the adjusted model. No significant associations were found for gender, race, age, or parent or guardian employment status in either model.

Table 4 shows logistic regression results for ever use of conventional cigarettes. In the adjusted logistic regression model, students with A grades had significantly lower odds of ever using conventional cigarettes compared to those with D and F grades (Adjusted Odds Ratio [AOR] = 0.15; *p* < 0.05). Parent or guardian employment was significantly associated with lower odds of cigarette use. Adolescents with employed parents had lower odds of cigarette use compared to those with unemployed parents (AOR = 0.40; *p* < 0.05). While students whose parents had a graduate degree or higher had reduced odds of cigarette use in the unadjusted model (Odds Ratio [OR] = 0.16; *p* < 0.05), this association was no longer statistically significant after adjustment. No significant associations were observed for gender, race, age, or living in a two-parent household in either model.

Table 5 shows logistic regression results for ever use of any tobacco product. In the adjusted logistic regression model, compared to students with D and F grades, those with A grades had significantly lower odds of any tobacco product use (Adjusted Odds Ratio [AOR] = 0.23; *p* < 0.05). While students with B grades also had reduced odds (AOR = 0.40), the association was marginally non-significant. No other variables were significantly associated with tobacco use in either the unadjusted or adjusted models.

## 5. Discussion

The most robust pattern emerged for e-cigarette use. Adolescents earning A, B, or C grades had significantly lower odds of ever using e-cigarettes than those with D or F grades. Similar, though slightly attenuated, trends were observed for conventional cigarettes and any tobacco product use, with A-grade students consistently demonstrating the lowest odds. In contrast, other factors such as gender, race, and age were not significantly associated with tobacco use in the adjusted models. Parental employment was inversely associated with conventional cigarette use, while the effects of parental education and household structure appeared significant in unadjusted models but diminished after covariate adjustment.

These results align with prior studies. A study [41] was conducted to examine whether the protective association between academic achievement and future susceptibility to tobacco use differs by ethnicity among adolescents in the United States. Using data from the Population Assessment of Tobacco and Health (PATH) study, this longitudinal analysis followed 3636 adolescents aged 12 to 17 who were never smokers at baseline, tracking them over a four-year period. Participants identified as Non-Latino White, African American, or Latino. The primary predictor was academic performance at baseline (Wave 1), measured by self-reported grades ranging from F to A+. The outcome was tobacco use susceptibility at follow-up (Wave 4), operationalized as openness to future tobacco use. Ethnicity was examined as a moderator, and models were adjusted for covariates including age, gender, parental education, and family structure. Results indicated that higher academic achievement was associated with lower tobacco use susceptibility four years later. However, this protective association was significantly weaker for African American and Latino youth compared to their Non-Latino White counterparts. Interaction terms confirmed that the strength of the association between school performance and tobacco susceptibility varied by ethnicity. These findings suggest that while academic success generally predicts lower susceptibility to tobacco use, its protective effect may be diminished for African American and Latino adolescents. This pattern may reflect broader contextual and structural challenges—such as school quality, neighborhood disadvantage, racial discrimination, and targeted marketing—that affect the usual behavioral benefits of educational success for minoritized youth. Further research is needed to investigate the mechanisms through which social context may shape health-risk behaviors among academically successful adolescents from racially and ethnically marginalized groups [41].

These findings may reflect multiple, interrelated mechanisms. Adolescents with lower academic performance might experience greater school-related stress, lower self-confidence, or less future orientation—factors that may increase vulnerability to coping behaviors like tobacco use [42,43,44,45]. Academic performance also likely reflects broader engagement with school, including connectedness to teachers, participation in structured activities, and adherence to behavioral expectations—all of which may be associated with reduced risk behaviors [46,47,48]. Furthermore, academic achievement may influence the type of peer networks adolescents form [49]. Students with lower grades may be more likely to affiliate with peers who engage in risk behaviors, increasing their exposure and susceptibility to tobacco use. In contrast, higher-achieving students may belong to peer groups where tobacco use is less socially accepted or prevalent [16].

It is also possible that adolescents who perform well academically possess greater health knowledge or are more responsive to anti-tobacco messages [50,51]. They may better understand the long-term health consequences of tobacco use, particularly concerning newer products like e-cigarettes, which are often perceived as less harmful. Additionally, academic performance may serve as a proxy for underlying cognitive, behavioral, or environmental factors, such as executive functioning, family organization, or school quality [52]—all of which may play roles in shaping adolescent behavior [53,54]. For Black adolescents specifically, experiences of structural inequality, school discipline disparities, and community-level disadvantage may contribute to both academic difficulties and higher health risks [8,20,55]. These contextual stressors may erode protective mechanisms and require more comprehensive and culturally responsive interventions.

The finding that parental employment is associated with lower odds of conventional cigarette use highlights the possible protective role of household economic stability. Employed parents may provide greater supervision, model healthier behaviors, or experience lower levels of household stress, all of which could help buffer adolescents from initiating tobacco use [56]. Although parental education was not significantly associated with tobacco use after adjustment, it may still influence adolescent outcomes indirectly through attitudes, knowledge, and communication about health risks [57].

A study analyzed data from 19,706 participants in Waves 9 (2017–2018) and 10 (2018–2019) of Understanding Society: The UK Household Longitudinal Study to examine the association between e-cigarette use and mental health over time. Using confirmatory factor analysis, linear mixed-effects models, and one-sample t-tests, the study found a significant interaction between time and e-cigarette initiation on mental health outcomes, including overall distress (b = 0.32; *p* < 0.001), social dysfunction and anhedonia (b = 0.36; *p* < 0.001), and loss of confidence (b = 0.24; *p* < 0.01). Individuals who began using e-cigarettes by Wave 10 reported worse mental health one year later, with small-to-moderate effect sizes, though no significant changes were observed in depression and anxiety. Limitations include self-reported data, lack of information on e-cigarette type, limited generalizability, and the non-experimental design. These findings suggest that e-cigarette initiation may be linked to declines in specific domains of mental health, highlighting the need for stronger regulation and public health interventions [58].

Recent intervention studies suggest that improving academic outcomes may also play a role in reducing tobacco use, including vaping, among youth. For instance, longitudinal analyses from the PATH study found that delaying or preventing e-cigarette initiation is associated with better academic performance later on [59]. School-based social and emotional learning (SEL) programs, which have been shown to strengthen academic performance, simultaneously reduce substance use by promoting skills like problem-solving and self-regulation. Innovative multi-level vaping prevention initiatives in school settings have further demonstrated potential in lowering e-cigarette initiation while also supporting students’ academic engagement [59]. Finally, broader community-based approaches—such as the Communities That Care framework—have consistently linked improvements in school bond and academic outcomes with reductions in tobacco use [59]. Taken together, these findings support the idea that holistic interventions targeting academic and psychosocial domains may have dual benefits: enhancing achievement while concurrently reducing tobacco use among underserved youth [60]. Future work should explore whether similar integrative strategies are especially effective in urban contexts experiencing both educational and tobacco-related inequities [40,61,62,63].

This study contributes uniquely to the existing literature by focusing on a predominantly low-income, urban Black adolescent population—a group that has been underrepresented in prior research on academic performance and tobacco use. While earlier studies have demonstrated a general inverse association between academic achievement and tobacco susceptibility, few have examined this relationship across specific tobacco product types (e-cigarettes vs. combustible cigarettes) within racially minoritized populations. Additionally, this study disaggregates by tobacco type and includes parental education, employment, and household structure as covariates, allowing for a more nuanced understanding of how family-level socioeconomic factors interact with academic performance in shaping adolescent tobacco use. This disaggregation highlights product-specific patterns and identifies differential associations between sociodemographic factors and tobacco subtypes, which can inform more targeted prevention strategies.

One surprising pattern in our data was the relatively high prevalence of tobacco use among students earning B and C grades, compared to those with D or F grades. This finding may reflect complex dynamics of school engagement and social identity. For example, students with mid-level academic performance might be more socially integrated than those with failing grades and thus more exposed to peer influence—a well-established risk factor for tobacco use initiation [64]. Additionally, students in the B/C range may experience pressures to maintain their performance, potentially leading to stress-related coping behaviors, including substance use [45]. Alternatively, adolescents with D or F grades may be more likely to be under stricter parental or institutional surveillance, limiting opportunities for substance use. Future research should explore whether patterns of peer affiliation, school connectedness, or parental monitoring vary across achievement levels and how these may moderate the relationship between academic performance and substance use.

The finding that parental employment was inversely associated with conventional cigarette use—but not with e-cigarettes or any tobacco product use—may reflect generational or cultural distinctions in how tobacco risk is perceived and discussed in households. Conventional cigarettes have long been stigmatized and are often the focus of health messaging, possibly making them more salient in health-related discussions between parents and children. Employed parents may be more likely to communicate norms against smoking traditional cigarettes, reflecting their own workplace policies or insurance incentives related to smoking cessation [65]. In contrast, e-cigarettes may be perceived as newer, less harmful, or less familiar by parents—especially among those with limited exposure to public health campaigns focused on vaping. This difference in parental awareness and attitudes may result in more effective socialization against cigarette smoking than against vaping. Additionally, the more visible and odorous nature of cigarette use may make it easier for employed parents to detect and intervene compared to the often stealthier use of e-cigarettes.

### 5.1. Implications

From a policy and practice standpoint, these results point to academic performance as a key social signal that can help identify adolescents who may be at higher risk of tobacco use [14]. Schools and public health programs might consider integrating tobacco prevention efforts with academic support services, targeting students who are struggling academically for more intensive health interventions. Screening for tobacco use in school-based or primary care settings could incorporate questions about academic performance to more effectively identify at-risk youth. At a broader level, these findings support the importance of investing in education equity initiatives, including funding for under-resourced schools, tutoring and mentoring programs, and culturally responsive curriculum which may help reduce both academic and behavioral disparities among Black youth.

Future research should aim to clarify the direction and mechanisms underlying these associations through longitudinal designs. Studies that track adolescents over time could assess whether academic difficulties precede tobacco initiation or whether early substance use contributes to declining academic performance. Further research should also incorporate measures of peer behavior, school climate, discrimination, and mental health to explore potential mediators. In addition, studies should examine these patterns in more diverse populations, including Latino and rural youth, to assess whether the observed associations hold across contexts. It would also be important to evaluate how educational and health systems can coordinate to support adolescents facing overlapping academic and behavioral challenges.

### 5.2. Limitations

This study has several limitations. Its cross-sectional design precludes causal inference, limiting our ability to determine the temporal order of academic performance and tobacco use [66], which may be subject to recall or social desirability bias [67]. In addition, the cross-sectional design precludes causal inference or temporal sequencing of academic performance and tobacco use. Our measures were based on single items, and such survey instruments may have limited reliability and validity. All measures are self-reported, and despite assurances of anonymity, social desirability bias may have influenced students’ disclosures about tobacco use. However, tobacco use and academic performance single-item measures have been validated in prior studies, particularly among similar populations [68,69]. The sample, though focused on a historically underserved population, was drawn from a single urban context, which may affect generalizability to other settings or populations. This study’s generalizability may be limited to similar urban, predominantly Black, low-income school populations, and it should be replicated in more diverse geographic and demographic settings. Additionally, while academic performance was categorized based on self-reported grades, this measure may not capture broader dimensions of educational engagement, such as school attendance, disciplinary actions, or standardized test scores [70]. While the introduction references several socio-structural factors (e.g., discrimination, neighborhood disadvantage), the study design and analysis did not explicitly integrate such variables. In addition, while the sample was drawn from multiple urban high schools, we were unable to account for clustering effects due to insufficient power for multilevel analysis. Furthermore, although we adjusted for key sociodemographic variables, unmeasured confounding factors—such as peer influence, mental health, or exposure to tobacco advertising—may have influenced both academic performance and tobacco use. Lastly, although the models adjusted for several sociodemographic variables, residual confounding by unmeasured factors such as parental tobacco use, household stress, or exposure to tobacco advertising may still exist.

While this study uses a cross-sectional design, it serves as a necessary first step in identifying population-specific associations that can inform future longitudinal work. Future studies should test hypotheses related to temporal ordering, such as whether poor academic performance leads to tobacco use or vice versa, and whether SEL interventions may disrupt this cycle. Additionally, prospective designs could assess mediators and moderators—such as perceived stress, discrimination, or school connectedness—to better understand the mechanisms by which academic performance influences tobacco initiation across time and setting.

Additionally, because the data were collected from students nested within multiple schools, we acknowledge the potential for school-level clustering effects. However, due to sample size limitations across each school/class, multilevel modeling was not feasible for the current analysis. Future studies should explore hierarchical data structures more fully to account for intra-school correlations and school-specific influences on both academic and behavioral health outcomes.

## 6. Conclusions

In conclusion, this study underscores the link between academic performance and tobacco use among Black adolescents. Adolescents who are excelling academically appear less likely to engage in tobacco use, particularly e-cigarette use, highlighting a potential avenue for early identification and intervention. These findings support the integration of academic and health promotion efforts and call for broader educational and economic policies that address the structural conditions shaping adolescent health behaviors. Reducing disparities in both education and health will likely require coordinated, equity-driven approaches across systems.

## Figures and Tables

**Table 1:** Descriptive statistics of the study sample (*n* = 604).

Variables	Total (*n* = 604)
Ever E-cigarette Use	
No	482 (79.8)
Yes	122 (20.2)
Ever Conventional Cigarette Use	
No	561 (92.9)
Yes	43 (7.1)
Ever Use of Any Tobacco Products	
No	452 (74.8)
Yes	152 (25.2)
Academic Performance (Grade)	
A	161 (26.7)
B	272 (45.0)
C	149 (24.7)
D & F	22 (3.6)
Gender	
Female	326 (54.0)
Male	260 (43.0)
Missing	18 (3.0)
Race	
Black	533 (88.3)
White	20 (3.3)
Others	51 (8.4)
Parent or Guardian Educational Attainment	
Less than high school diploma	75 (12.4)
High school graduation	215 (35.6)
Some college	129 (21.4)
College graduation	78 (12.9)
Graduate degree or higher	104 (17.2)
Missing	3 (0.5)
Parent or Guardian Employment	
No	87 (14.4)
Yes	515 (85.3)
Missing	2 (0.3)
Living in Double-Parent Household	
No	378 (62.6)
Yes	225 (37.2)
Missing	1 (0.2)
	Mean (SD)
Age (Years)	16.1 (1.5)

**Table 2:** Descriptive statistics of the study sample by academic performance (grade).

Variables	Academic Performance (Grade)				Total (*n* = 604)
	A	B	C	D & F	
Ever E-cigarette Use ***					
No	139 (28.8)	220 (45.6)	112 (23.2)	11 (2.3)	482
Yes	22 (18.0)	52 (42.6)	37 (30.3)	11 (9.0)	122
Ever Conventional Cigarette Use *					
No	157 (28.0)	250 (44.6)	136 (24.2)	18 (3.2)	561
Yes	4 (9.3)	22 (51.2)	13 (30.2)	4 (9.3)	43
Ever Use of Any Tobacco Products ***					
No	136 (30.1)	208 (46.0)	97 (21.5)	11 (2.4)	452
Yes	25 (16.5)	64 (42.1)	52 (34.2)	11 (7.2)	152
Gender					
Female	98 (30.1)	136 (41.7)	78 (23.9)	14 (4.3)	326
Male	59 (22.7)	129 (49.6)	65 (25.0)	7 (2.7)	260
Missing	4 (22.2)	7 (38.9)	6 (33.3)	1 (5.6)	18
Race					
Black	139 (26.1)	238 (44.6)	137 (25.7)	19 (3.6)	533
White	10 (50.0)	6 (30.0)	2 (10.0)	2 (10.0)	20
Others	12 (23.5)	28 (54.9)	10 (19.6)	1 (2.0)	51
Parent or Guardian Educational Attainment *					
Less than high school diploma	12 (16.0)	40 (53.3)	19 (25.4)	4 (5.3)	75
High school graduation	55 (25.6)	85 (39.5)	63 (29.3)	12 (5.6)	215
Some college	41 (31.8)	59 (45.7)	26 (20.2)	3 (2.3)	129
College graduation	22 (28.2)	31 (39.7)	22 (28.2)	3 (3.9)	78
Graduate degree or higher	30 (28.9)	56 (53.8)	18 (17.3)	-	104
Missing	1 (33.3)	1 (33.3)	1 (33.3)	-	3
Parent or Guardian Employment					
No	25 (28.7)	35 (40.2)	21 (24.2)	6 (6.9)	87
Yes	136 (26.4)	235 (45.6)	128 (24.9)	16 (3.1)	515
Missing	-	2 (100.0)	-		2
Living in Double-Parent Household					
No	97 (25.7)	166 (43.9)	99 (26.2)	16 (4.2)	378
Yes	63 (28.0)	106 (47.1)	50 (22.2)	6 (2.7)	225
Missing	1 (100.0)	-	-	-	
	Mean (SD)	Mean (SD)	Mean (SD)	Mean (SD)	Mean (SD)
Age (Years) *	16.2 (1.3)	16 (1.4)	16.2 (1.6)	16.8 (1.8)	16.1 (1.5)

Note. *** *p* < 0.001; * *p* < 0.05. Abbreviation: SD = Standard Deviation.

**Table 3:** Logistic regression for electronic cigarette use.

Variables	Ever Electronic Cigarette Use	
	Unadjusted OR (95% CR)	Adjusted OR (95% CR)
Academic Performance (Grade)		
D & F	Ref.	Ref.
C	0.36 * (0.15, 0.89)	0.38 * (0.14, 0.99)
B	0.26 ** (0.11, 0.62)	0.29 * (0.11, 0.75)
A	0.17 *** (0.07, 0.44)	0.19 * (0.07, 0.50)
Gender		
Female	Ref.	Ref.
Male	0.81 (0.53, 1.22)	0.87 (0.57, 1.34)
Race		
White	Ref.	Ref.
Black	0.57 (0.21, 1.53)	0.48 (0.17, 1.39)
Others	0.64 (0.20, 2.06)	0.59 (0.17, 2.03)
Age (Years)	1.00 (0.87, 1.15)	0.99 (0.85, 1.14)
Parent or Guardian Educational Attainment		
Less than high school diploma	Ref.	Ref.
High school graduation	0.66 (0.36, 1.21)	0.70 (0.37, 1.33)
Some college	0.71 (0.37, 1.37)	0.77 (0.38, 1.56)
College graduation	0.51 (0.24, 1.12)	0.57 (0.24, 1.33)
Graduate degree or higher	0.47 * (0.22, 0.97)	0.55 (0.25, 1.21)
Parent or Guardian Employment		
No	Ref.	Ref.
Yes	1.05 (0.60, 1.87)	1.26 (0.67, 2.36)
Living in Double-Parent Household		
No	Ref.	Ref.
Yes	0.65 * (0.42, 0.99)	0.75 (0.47, 1.19)

Note. *** *p* < 0.001; ** *p* < 0.01; * *p* < 0.05. Abbreviation: OR = Odds Ratio.

**Table 4:** Logistic regression for conventional cigarette use.

Variables	Ever Conventional Cigarette Use	
	Unadjusted OR (95% CR)	Adjusted OR (95% CR)
Academic Performance (Grade)		
D & F	Ref.	Ref.
C	0.43 (0.13, 1.46)	0.51 (0.14, 1.87)
B	0.40 (0.12, 1.27)	0.52 (0.15, 1.81)
A	0.11 ** (0.03, 0.50)	0.15 * (0.03, 0.67)
Gender		
Female	Ref.	Ref.
Male	1.09 (0.58, 2.06)	1.11 (0.57, 2.16)
Race		
White	Ref.	Ref.
Black	0.67 (0.15, 3.00)	0.58 (0.11, 2.90)
Others	0.77 (0.13, 4.55)	0.80 (0.12, 5.38)
Age (Years)	1.01 (0.81, 1.25)	0.98 (0.78, 1.22)
Parent or Guardian Educational Attainment		
Less than high school diploma	Ref.	Ref.
High school graduation	0.86 (0.36, 2.04)	1.10 (0.43, 2.82)
Some college	0.63 (0.23, 1.70)	1.00 (0.35, 2.90)
College graduation	0.45 (0.13, 1.57)	0.74 (0.20, 2.78)
Graduate degree or higher	0.16 * (0.03, 0.80)	0.28 (0.05, 1.43)
Parent or Guardian Employment		
No	Ref.	Ref.
Yes	0.40 * (0.20, 0.81)	0.40 * (0.18, 0.88)
Living in Double-Parent Household		
No	Ref.	Ref.
Yes	0.80 (0.41, 1.55)	0.95 (0.46, 1.97)

Note. ** *p* < 0.01; * *p* < 0.05. Abbreviation: OR = Odds Ratio.

**Table 5:** Logistic regression for use of any tobacco products.

Variables	Ever Use of Any Tobacco Products	
	Unadjusted OR (95% CR)	Adjusted OR (95% CR)
Academic Performance (Grade)		
D & F	Ref.	Ref.
C	0.54 (0.22, 1.32)	0.67 (0.26, 1.71)
B	0.31 ** (0.13, 0.74)	0.40 (0.16, 1.02)
A	0.18 *** (0.07, 0.47)	0.23 * (0.08, 0.60)
Gender		
Female	Ref.	Ref.
Male	0.84 (0.57, 1.22)	0.86 (0.58, 1.29)
Race		
White	Ref.	Ref.
Black	0.78 (0.29, 2.06)	0.64 (0.23, 1.82)
Others	0.80 (0.25, 2.51)	0.74 (0.22, 2.50)
Age (Years)	1.08 (0.96, 1.23)	1.08 (0.95, 1.24)
Parent or Guardian Educational Attainment		
Less than high school diploma	Ref.	Ref.
High school graduation	0.70 (0.40, 1.24)	0.75 (0.41, 1.39)
Some college	0.72 (0.39, 1.33)	0.86 (0.44, 1.68)
College graduation	0.52 (0.25, 1.07)	0.58 (0.26, 1.28)
Graduate degree or higher	0.51 (0.26, 1.00)	0.66 (0.32, 1.36)
Parent or Guardian Employment		
No	Ref.	Ref.
Yes	0.87 (0.52, 1.45)	0.98 (0.56, 1.72)
Living in Double-Parent Household		
No	Ref.	Ref.
Yes	0.71 (0.48, 1.06)	0.83 (0.55, 1.28)

Note. *** *p* < 0.001; ** *p* < 0.01; * *p* < 0.05. Abbreviation: OR = Odds Ratio.

## Data Availability

Data are available upon request due to IRB.

## Abbreviations

**Abbreviation**	**Full Term**
AOR	Adjusted Odds Ratio
CBPR	Community-Based Participatory Research
CEASE	Communities Engaged and Advocating for a Smoke-free Environment
CI	Confidence Interval
D & F	Grades D and F (combined reference category)
DMN	Default Mode Network
DL-PFC or dlPFC	Dorsolateral Prefrontal Cortex
FDR	False Discovery Rate
fMRI	Functional Magnetic Resonance Imaging
GMM	Growth Mixture Modeling
ICA	Independent Component Analysis
IRB	Institutional Review Board
LMEM	Linear Mixed-Effects Model
MID	Monetary Incentive Delay (Task)
mPFC	Medial Prefrontal Cortex
NAcc	Nucleus Accumbens
N-O-T	Not On Tobacco (program)
NRT	Nicotine Replacement Therapy
OR	Odds Ratio
PCC	Posterior Cingulate Cortex
PCA	Principal Component Analysis
PPI	Psychophysiological Interaction
RCT	Randomized Controlled Trial
ROI	Region of Interest
SD	Standard Deviation
SEL	Social and Emotional Learning
SEM	Structural Equation Modeling
SES	Socioeconomic Status
STROBE	Strengthening the Reporting of Observational Studies in Epidemiology
VTA	Ventral Tegmental Area
